# Pharmacological and functional characterization of the first small molecule TREM2 agonist, VG‐3927, for the treatment of Alzheimer’s disease

**DOI:** 10.1002/alz.089318

**Published:** 2025-01-09

**Authors:** Christian Mirescu, Borislav Dejanovic, Kelley C. Larson, Beril Kiragasi, Frederick W. Gergits, Matthew D. Figley, Abigail J. Renoux, Liyue Huang, Bhaumik A. Pandya, Jonathan B. Houze, Evan A. Thackaberry, David L. Gray

**Affiliations:** ^1^ Vigil Neuroscience, Inc, Watertown, MA USA

## Abstract

**Background:**

TREM2 is a lipid‐sensing receptor expressed by microglial sub‐populations within neuropathological microenvironments, whose downstream signaling promotes microglial survival, plasticity, and migration. Multiple loss‐of‐function variants strongly implicate TREM2 as a key regulator of Alzheimer’s disease (AD) risk. Accordingly, TREM2 antibodies are currently in development to evaluate the therapeutic potential of TREM2 agonism in neurodegenerative diseases. Small molecule TREM2 agonist could represent a significant advancement for patients by providing potential key advantages in route of administration, superior brain penetration, and alternative mechanisms of action versus monoclonal antibodies. Herein, we share recent *in vitro* and *in vivo* preclinical data identifying VG‐3927, the first and only small molecule TREM2 agonist in clinical development as a potential disease‐modifying therapeutic.

**Methods:**

The agonist pharmacology of VG‐3927 was determined across an array of human cellular systems. Human iPSC‐derived microglia, astrocytes and neurons tri‐cultures were utilized to measure changes in microglia function and impact on neurodegeneration biomarkers in response to pharmacological and genetic modulation. Additionally, a humanized TREM2 amyloidosis mouse model (hTREM2‐5xFAD) was employed to determine the impact of VG‐3927 on AD‐associated neuropathological endpoints.

**Results:**

Pharmacologic data suggest VG‐3927 is a highly potent, selective and brain penetrant TREM2 agonist with a favorable functional profile. In human tri‐cultures, VG‐3927 promoted anti‐inflammatory microglia activation and suppressed a broad spectrum of neurodegeneration‐associated biomarkers. Mutagenesis mapping studies demonstrated that the activity of VG‐3927 is on‐target, and results in functional effects via the coordinated clustering of TREM2. Following only 6 weeks of oral dosing in humanized TREM2 plaque‐bearing mice, VG‐3927 also reduced pathological forms of Aβ, insoluble ApoE levels, and peri‐plaque dystrophic neurites. Additional PKPD studies in non‐human primates further confirmed the brain penetrance and pharmacological activity in CNS.

**Conclusions:**

VG‐3927 represents the first potent, selective small molecule TREM2 agonist able to favorably tune human microglia activation and impart a broadly neuroprotective profile across preclinical model systems. Initial PKPD studies have confirmed brain penetrance and pharmacological activity in CNS; an ongoing clinical study aims to further interrogate the potential of VG‐3927 as an AD modifying therapeutic with transformative potential as an orally bioavailable treatment.

